# The Potential Clinical Utility of the Customized Large Language Model in Gastroenterology: A Pilot Study

**DOI:** 10.3390/bioengineering12010001

**Published:** 2024-12-24

**Authors:** Eun Jeong Gong, Chang Seok Bang, Jae Jun Lee, Jonghyung Park, Eunsil Kim, Subeen Kim, Minjae Kimm, Seoung-Ho Choi

**Affiliations:** 1 Department of Internal Medicine, Hallym University College of Medicine, Chuncheon 24253, Republic of Korea; gong-eun@hanmail.net; 2 Institute for Liver and Digestive Diseases, Hallym University, Chuncheon 24253, Republic of Korea; 3 Institute of New Frontier Research, Hallym University College of Medicine, Chuncheon 24253, Republic of Korea; iloveu59@hallym.or.kr; 4 Department of Anesthesiology and Pain Medicine, Hallym University College of Medicine, Chuncheon 24253, Republic of Korea; 5 Meninblox, Inc., Gwangju 61008, Republic of Korea; benjamin@meninblox.com (J.P.); lucy@meninblox.com (E.K.); anna@meninblox.com (S.K.); 6 Department of Plastic Art, Tech University of Korea, Siheung 15073, Republic of Korea; plastic@tukorea.ac.kr; 7 College of Liberal Arts Faculty of Basic Liberal Art, Hansung University, Seoul 02876, Republic of Korea; jcn99250@naver.com

**Keywords:** large language model, artificial intelligence, gastroenterology

## Abstract

**Background:** The large language model (LLM) has the potential to be applied to clinical practice. However, there has been scarce study on this in the field of gastroenterology. Aim: This study explores the potential clinical utility of two LLMs in the field of gastroenterology: a customized GPT model and a conventional GPT-4o, an advanced LLM capable of retrieval-augmented generation (RAG). **Method:** We established a customized GPT with the BM25 algorithm using Open AI’s GPT-4o model, which allows it to produce responses in the context of specific documents including textbooks of internal medicine (in English) and gastroenterology (in Korean). Also, we prepared a conventional ChatGPT 4o (accessed on 16 October 2024) access. The benchmark (written in Korean) consisted of 15 clinical questions developed by four clinical experts, representing typical questions for medical students. The two LLMs, a gastroenterology fellow, and an expert gastroenterologist were tested to assess their performance. **Results:** While the customized LLM correctly answered 8 out of 15 questions, the fellow answered 10 correctly. When the standardized Korean medical terms were replaced with English terminology, the LLM’s performance improved, answering two additional knowledge-based questions correctly, matching the fellow’s score. However, judgment-based questions remained a challenge for the model. Even with the implementation of ‘Chain of Thought’ prompt engineering, the customized GPT did not achieve improved reasoning. Conventional GPT-4o achieved the highest score among the AI models (14/15). Although both models performed slightly below the expert gastroenterologist’s level (15/15), they show promising potential for clinical applications (scores comparable with or higher than that of the gastroenterology fellow). **Conclusions:** LLMs could be utilized to assist with specialized tasks such as patient counseling. However, RAG capabilities by enabling real-time retrieval of external data not included in the training dataset, appear essential for managing complex, specialized content, and clinician oversight will remain crucial to ensure safe and effective use in clinical practice.

## 1. Introduction

The integration of artificial intelligence (AI) into healthcare has been an evolving process over the past few decades, beginning with simple decision-support systems and evolving into the more sophisticated large language models (LLMs) we see today. These models, like OpenAI’s GPT-4o (Generative Pre-trained Transformer, OpenAI Inc., San Francisco, CA, USA) have demonstrated the ability to analyze vast amounts of medical data, offering new opportunities for improving diagnostic accuracy, enhancing patient care, and supporting clinical decision-making across multiple medical specialties, including radiology, oncology, and gastroenterology [1].

With the increasing availability of medical literature and clinical data, healthcare professionals often face challenges in efficiently accessing relevant information. LLMs have shown promising potential in automating text summarization, information retrieval, and knowledge synthesis [1,2,3]. In the field of gastroenterology, timely and accurate access to clinical knowledge is essential for both education and clinical decision-making. However, the clinical utility of such models in real-world settings requires further evaluation, especially in scenarios involving specialized terminology and context-specific information [4,5].

Recent advancements in AI technology have enabled LLMs to go beyond simple text analysis and retrieval, entering a new era in which they can support decision-making processes directly in complex clinical environments. For example, AI-based models have shown remarkable performance in imaging analysis, such as radiology, by identifying anomalies with a high degree of precision [2]. Similarly, in oncology, AI models assist in predicting cancer prognosis by analyzing genetic profiles and clinical features [3]. These applications demonstrate the expansive possibilities of LLMs in transforming various medical domains, and gastroenterology is no exception. Gastroenterology, being a field that requires nuanced interpretation of clinical symptoms and endoscopic findings, stands to benefit significantly from LLMs’ capabilities to synthesize large volumes of specialized information quickly [6].

Moreover, the need for efficient data synthesis is underscored by the rapid growth of the gastroenterological literature. Practitioners must stay informed about developments in diagnostic criteria, treatment guidelines, and emerging research findings. However, the sheer volume of information often makes this task daunting. LLMs, equipped with capabilities for natural language processing, offer a practical solution by summarizing research articles, extracting key insights, and even answering specific clinical questions [7]. Such automation can alleviate the cognitive burden on healthcare professionals, thereby improving their ability to provide patient-centered care.

A GPT is a type of AI model that trains on large amounts of text data to perform a variety of text generation tasks, and they specialize in text generation. ChatGPT has natural conversation ability with powerful language generation capabilities because it was built based on GPT. A customized GPT is a model that has been further trained for a specific purpose and is more knowledgeable in a particular field, providing more accurate information and enabling it to provide different services tailored to needs.

In recent years, it has become possible to customize LLMs for specific tasks or domains by fine-tuning them on specialized datasets. This customization allows for the creation of models that are tailored to the unique requirements of specific fields, such as gastroenterology, thereby improving the accuracy and relevance of their outputs. For instance, a customized LLM trained on gastroenterology textbooks and research papers can offer more precise responses compared with a general-purpose model. This approach enhances the practical utility of LLMs in clinical environments in which specialized knowledge is crucial.

Additionally, LLMs have now evolved to include real-time Internet access capabilities, known as retrieval-augmented generation (RAG). This functionality enables models to retrieve and incorporate the most up-to-date information available on the Internet, providing responses that reflect the current research and clinical guidelines. However, the question of whether customized LLMs or RAG-enabled models offer superior performance remains unanswered. Each approach has distinct advantages: customized LLMs benefit from domain-specific training, while RAG-enabled models provide real-time, dynamic knowledge retrieval. The optimal approach may depend on the specific clinical context and the type of information required.

A major challenge in clinical education and practice lies in quickly identifying and synthesizing key information from large volumes of unstructured data. Tools that integrate PDF text extraction, efficient search algorithms, and language models capable of summarization can potentially transform how healthcare providers access and use information. In educational settings, such models can assist students and trainees by quickly retrieving relevant knowledge [1,7]. In addition, integrating AI-driven solutions like LLMs into the medical curriculum can enhance learning experiences by providing instant access to authoritative knowledge bases, thereby bridging the gap between theoretical knowledge and clinical practice.

This study focuses on the performance of a customized GPT model and conventional GPT-4o model in comparison with a gastroenterology fellow and an expert gastroenterologist when faced with clinically relevant exam questions. The comparison aims to evaluate the model’s capacity to handle domain-specific content and assess how changes in terminology (Korean to English) influence its accuracy. Additionally, by identifying areas in which the LLM struggled, such as inferential reasoning and non-textbook knowledge, this research aims to provide insights into the model’s strengths and limitations, shedding light on its potential role as a supplementary educational and decision-making tool.

## 2. Methods

This study was approved by the institutional review board of Chuncheon Sacred Heart hospital (No. 2024-01-005).

### 2.1. System Design and Workflow of Customized LLM

This study utilizes a customized LLM-based system for processing and extracting relevant data from PDF files. The system integrates several components including the PyMuPDF Python library (PyMuPDF 1.25.1) for text and image extraction, the BM25 (or Okapi BM25) algorithm for text similarity search, and OpenAI’s GPT-4o for summarization. The following describes the workflow and technical details.

### 2.2. Dataset Preprocessing for Customized LLM

The training dataset used to create the customized embedding model consisted of 18 PDFs with images and texts extracted from an average of 240 pages. Preprocessing of PDF files of specialized books related to the digestive system was performed using Python’s PyMuPDF (fitz) library (PyMuPDF 1.25.1). In this process, text and images were extracted from each PDF file, and the extracted data were structured so that they could be used for analysis and model learning. First, each PDF file was loaded page by page using the fitz library, and then images were extracted from each page and saved with a unique file name according to the page number and image index. In the case of multiple PDF files, parallel processing technology was applied using the concurrent.futures module to shorten the processing time. The detailed data preprocessing method is described below.

### 2.3. PDF Processing and Optimization

PDF files of an internal medicine textbook (written in English and in Korean, Joseph Loscalzo, Anthony Fauci, Dennis Kasper, Stephen Hauser, Dan Longo, J. Larry Jameson et al. Harrison’s Principles of Internal Medicine 19th edition, McGraw Hill, Corp., New York, NY, USA) [8] and a gastroenterology textbook (written in Korean, Chung—Yong Kim et al. Chung—Yong Kim’s Gastroenterology textbook, 4th edition, ilchokak Co., Ltd., Seoul, Republic of Korea) [9] were processed using PyMuPDF (fitz) on a page-by-page basis. The extracted images were saved in the format ‘filename_page.jpeg’ using the extract_images_from_pdf() function. Text extraction for all the PDFs was handled by the extract_text_from_multiple_pdfs() function, with parallel processing implemented using concurrent.futures.

### 2.4. Parallel Processing and Search Algorithm

For performance optimization, multiple PDF files were processed simultaneously with concurrent.futures.ThreadPoolExecutor. The BM25 algorithm was used to search the extracted text and identify relevant pages based on user queries. The find_relevant_pages_bm25() function tokenized the text and provided relevance scores. The top-ranked pages were summarized using GPT-4, with extracted images displayed alongside summaries in a Streamlit-based interface (https://github.com/SonicWarrior1/pdfchat (accessed on 8 November 2024)).

## 3. Experimental Method for the Customized LLM

A Python-based PDF processing code was used to process and analyze text and images of specialized books related to the digestive system. After performing text extraction and image analysis for each file, answers to the user’s questions were generated using the BM25 algorithm and the GPT-4o model. The task of finding pages related to the given question was performed using the BM25 algorithm. After tokenizing the user-entered question, the most relevant pages were found based on the BM25 score and used to generate answers to the questions using OpenAI’s GPT-4o model. A function to summarize the content of the extracted pages using the GPT-4o model was also provided. The response time was measured using Python’s time module. The results were provided to the user through the Streamlit interface. In addition, the visual information provided in the PDF was also provided by outputting the extracted images together to the Streamlit interface.

## 4. Experimental Method for the Conventional GPT-4o

Conventional GPT-4o is an advanced LLM capable of retrieval-augmented generation (RAG). By enabling the real-time extraction of external data that were not included in the training dataset, this approach improves classic LLMs by producing answers that incorporate the most recent information [7]. By using this method, hallucinations can be avoided, and a wider range of knowledge can be incorporated into the model. We accessed this model (https://chatgpt.com/) accessed on 5 October 2024.

## 5. Experimental Setup and Benchmarking

The performance metrics were evaluated on two hardware setups: a laptop (Intel core Ultra 7 155H CPU, Intel Arc Graphics GPU, 32GB RAM (Santa Clara, CA, USA)) and a desktop computer system (Intel i7-10700k CPU, RTX 2070 super 8GB GPU, 32GB RAM (Santa Clara, CA, USA)) with different configurations. Additionally, a benchmark exam consisting of 15 medical questions was created by four clinical expert gastroenterologists, with standardized Korean medical terminology. All of them had experience in taking the Korean medical board examination or the Korean Society of Gastrointestinal Endoscopy license examination, and they created the questions in the same way as we did for this exam. Typical exam questions for medical students were established and were validated by all experts before being used for testing. Benchmark questions consisted of 10 knowledge-based and 5 judgment-based questions. Each question was written in Korean and consisted of five multiple-choice answers. The representative knowledge-based and judgment-based questions are translated into English below.

Representative knowledge-based question: A 32-year-old woman presents with acute abdominal pain. She reported eating sushi the day before. An esophagogastroduodenoscopy was performed on the day of the visit and was diagnosed as anisakiasis. Which of the following is most likely to be indicated? | Proton pump inhibitor treatment | Albendazole treatment | Oral steroids treatment| Praziquantel treatment | Endoscopic removal using biopsy forceps.

Representative judgment-based question: A 70-year-old man presents to the emergency department with bloody stool. His blood pressure is 80/50 mm Hg, pulse rate is 110 beats/min, and temperature is 36.9 degrees Celsius. Which test is most likely to be ordered next? | 99mTc-pertechnate scan | capsule endoscopy | esophagogastroscopy | abdominal computed tomography | abdominal arterial angiography.

The benchmark questions were presented to the customized GPT model, conventional GPT-4o model, a gastroenterology fellow, and an expert gastroenterologist, and a comparative evaluation was made.

## 6. Results

The customized GPT model answered 8 questions correctly out of 15, while the gastroenterology fellow answered 10 correctly. When the standardized Korean medical terms were replaced with English terminology, the LLM’s performance improved, answering two additional knowledge-based questions correctly, matching the fellow’s score (10 out of 15). However, judgment-based questions remained a challenge for the model.

Even with the implementation of ‘Chain of Thought’ prompt engineering, the customized GPT did not achieve improved reasoning. Incorrect answers from the customized GPT model were primarily related to inferential reasoning and recent medical knowledge not presented in textbooks.

**Figure 1:**
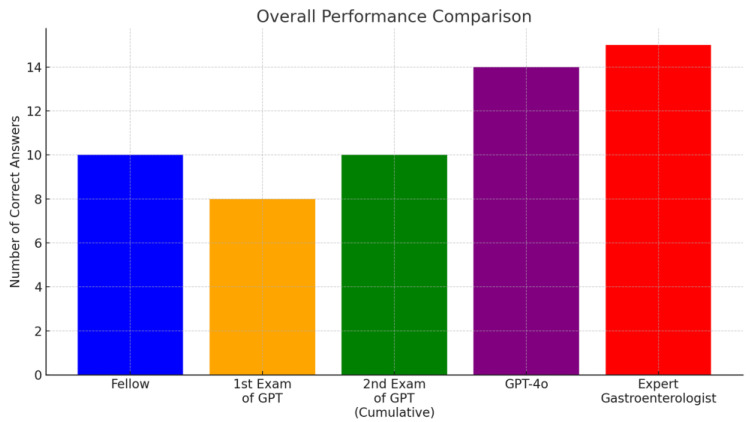
Overall performance comparison.

**Figure 2:**
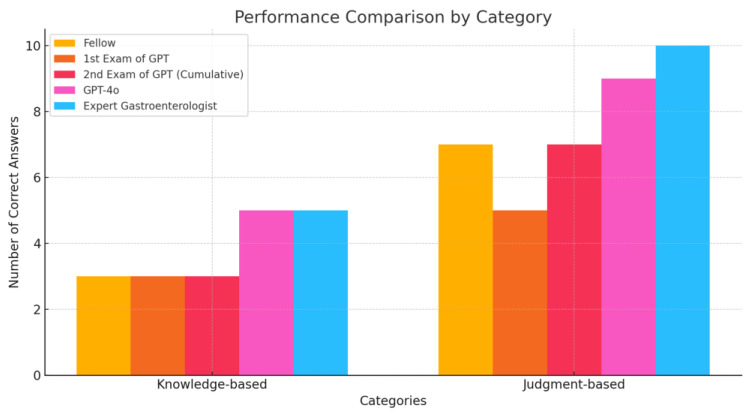
Performance comparison by category.

Conventional GPT-4o achieved the highest score among the AI models (14/15). Although both models performed slightly below the expert gastroenterologist’s level (15/15), they show promising potential for clinical applications (scores comparable with or higher than that of the gastroenterology fellow). Table 1 shows the summary of the performances. Figure 1 describes the overall performance comparison, and Figure 2 shows the performance comparison by category.

The average response time per question on a laptop computer was 293.45 s (under an Internet speed of 290 Mbps), and the average response time per issue on a desktop computer was 284.58 s (under an Internet speed of 480 Mbps).

## 7. Discussion

The findings of this study highlight the potential of LLMs in the field of gastroenterology. Both the customized GPT and GPT-4o models demonstrated performance on par with or exceeding that of a gastroenterology fellow, suggesting their utility in specialized clinical settings. However, the slightly lower performance compared with the expert gastroenterologist indicates that, while LLMs can provide valuable support, they cannot fully replace human expertise now.

A key advancement in AI that makes this possible is RAG. Unlike traditional models that rely solely on pre-existing knowledge, RAG-enabled models like GPT-4o can dynamically retrieve the latest medical data, including research articles, clinical trials, and updated guidelines. This capability is particularly important in fields like gastroenterology, in which treatment protocols and best practices are frequently updated based on new evidence. Without real-time access to updated knowledge, AI models may lag behind in accuracy, potentially compromising patient care [7,10].

Furthermore, recent studies have shown that the customization of LLMs plays a crucial role in enhancing their applicability in specialized fields. Customized models that are fine-tuned on domain-specific data can provide more accurate and contextually relevant responses. For instance, Gorelik Y et al. [11] established a customized GPT model to provide guideline-based management advice for pancreatic cysts, aligning with expert recommendations in 87% of scenarios. Such results demonstrate that fine-tuning can help bridge the performance gap between AI models and medical experts, especially in specific domains.

However, the debate on whether customized models or RAG-enabled generalized models are superior remains unresolved. While RAG provides the advantage of real-time access to current data, customized models offer consistency and reliability by relying on thoroughly validated datasets. A balance between these two approaches could potentially yield the best outcomes, combining the strengths of specialized knowledge with the adaptability of real-time information retrieval. This hybrid approach may be particularly beneficial for complex, evolving medical fields like gastroenterology, where both up-to-date knowledge and deep domain understanding are crucial [12,13,14,15].

Another consideration is the role of clinician oversight in ensuring the appropriate use of LLMs. Although LLMs like GPT-4o demonstrate promising potential, their integration into clinical practice must involve careful validation and continuous monitoring. Clinician oversight is particularly crucial for judgment-based scenarios in which AI models may lack the nuanced understanding needed for accurate clinical decision-making. In practice, LLMs can serve as a supplementary tool, providing initial recommendations or summarizing information, which clinicians can then verify and interpret in the context of individual patient needs [15].

Additionally, while LLMs have the ability to assist with patient counseling and streamline information retrieval, there are still limitations in their capacity to perform complex clinical reasoning tasks. This study found that judgment-based questions, which often require inferential thinking and the ability to weigh multiple clinical factors, were particularly challenging for the LLMs. Addressing this limitation will require further advancements in AI model training, possibly involving more complex datasets that include real-world clinical scenarios and decision pathways [16,17].

In the context of the detailed results of this study, the superior performance of GPT-4o (compared with customized GPT) emphasizes the need for real-time access to an extensive knowledge base, particularly for complex, evolving domains such as gastroenterology. Models limited to static data, like the customized GPT, may struggle to achieve the same level of accuracy, highlighting the limitations of training on a finite set of resources. Although the customized GPT also incorporates the concept of RAG, which allows it to produce responses in specialized PDF documents, its access to a wider range of data than that available to the conventional GPT produced the best performance in this study [1]. The conventional GPT-4o provided accurate clinical reasoning and commentary, whereas the customized GPT referred to paragraphs, sentences, and figures only in the PDF files. In the representative example of the judgment-based question above, the customized GPT searched for the chief complaint of hematochezia and concluded that a pancreatic tumor was the first suspicion and a CT scan was needed to characterize the tumor, whereas the conventional GPT concluded that the hematochezia, hypotension (80/50 mmHg), and tachycardia (110 beats/min) in the 70-year-old man strongly suggested digestive tract bleeding. Conventional GPT-4o presented correct reasoning that, in cases of massive bleeding, it is not uncommon for the bloody stool to be due to upper gastrointestinal bleeding (esophagus, stomach, duodenum) and the first test to be performed is esophagogastroscopy to confirm the presence of upper gastrointestinal bleeding for the early identification of the bleeding site and endoscopic hemostasis, especially in an emergency situation.

Another important finding from this study is the language issue with LLMs. This study showed that LLMs performed better in answering questions and scored higher when asked in English rather than in Korean. Yeo YH et al. (2023) also assessed ChatGPT and GPT-4’s ability to comprehend and respond to cirrhosis-related questions in English, Korean, Mandarin, and Spanish, addressing language barriers that may impact patient care [18]. In their study, GPT-4 showed a marked improvement in the proportion of comprehensive and correct answers compared with ChatGPT across all four languages, and GPT-4 demonstrated enhanced accuracy and avoided erroneous responses evident in ChatGPT’s output, indicating that LLMs’ language-specific responses are something that must be addressed in the future.

Previous studies utilizing LLMs have also investigated potential clinical applications. In order to assess reliability, the majority of studies [12,13,14,15,16,17,18,19,20,21,22,23,24,25] asked LLMs about common or specific medical knowledge, such as management of gastroesophageal reflux disease, nutrition-related questions to inflammatory bowel disease, screening and surveillance intervals for colonoscopies, or board examination tests. Expert endoscopists or gastroenterologists rated the performance of the LLM in these trials, and the majority of the investigations demonstrated practical application. All of these studies, however, used conventional LLMs that were not customized or modified to the specified task in contrast to our study.

The integration of LLMs into clinical settings introduces significant regulatory and ethical considerations that must be addressed to ensure their safe and effective use. Regulatory challenges primarily revolve around ensuring compliance with medical device standards, data protection regulations, and guidelines on clinical decision-support systems. For instance, the use of patient data in training models raises concerns about privacy and adherence to the local laws. Ensuring the transparency and reproducibility of LLM outputs is another key regulatory requirement to foster trust and reliability in clinical practice. From an ethical standpoint, the potential for algorithmic bias poses risks, particularly in underrepresented patient populations, which could lead to disparities in care. Additionally, the deployment of LLMs necessitates a robust framework for accountability, delineating clear boundaries between clinician and AI responsibilities to prevent over-reliance on automated systems. These considerations underscore the importance of clinician oversight, ongoing validation, and alignment with established medical and ethical standards as LLMs are progressively adopted in gastroenterology and beyond.

The clinical applications of LLMs in gastroenterology are promising. They could assist with patient counseling, streamline information retrieval, and provide decision support for healthcare professionals [1,2,3,7]. However, it is essential to recognize that these models are not infallible. Errors in judgment-based reasoning observed in this study demonstrate the need for continuous clinician oversight to ensure accuracy and safety. Furthermore, the models’ ability to explain their reasoning and provide transparent responses will be critical for gaining trust in clinical environments [7,10]. While LLMs like GPT-4o offer significant potential for use in gastroenterology, their integration into clinical practice must be accompanied by careful validation, RAG capabilities, and appropriate oversight. With these considerations, LLMs can serve as valuable tools to enhance healthcare delivery and improve patient outcomes [16,17,18,19].

## 8. Conclusions

This study explores the potential clinical utility of two LLMs in the field of gastroenterology: a customized GPT model and GPT-4o, an advanced LLM capable of RAG. While the customized GPT was trained on several gastroenterology textbooks, GPT-4o utilized RAG to access a broader knowledge base in real time. Both models demonstrated performances comparable with or superior to that of a gastroenterology fellow, with GPT-4o achieving the highest score among the AI models. Although both models performed slightly below the expert gastroenterologist’s level, these results indicate potential clinical utility, especially in supporting patient counseling. However, RAG appears essential for managing the breadth and depth of specialized content, and clinician oversight will be crucial for ensuring reliable outcomes in clinical practice.

## Figures and Tables

**Table 1:** The summary of performance for the benchmark test.

Category	Gastroenterology Fellow	Customized GPT (1st Test)	Customized GPT (2nd Test)	Conventional GPT-4o	Expert Gastroenterologist
Judgment based (n = 5)	3	3	0	5	5
Knowledge based (n = 10)	7	5	10	9	10
Total (n = 15) (correct answer)	10	8	10	14	15

GPT, generative pre-trained transformer.

## Data Availability

All the data are accessible and available upon request from the corresponding author. Access to data: all investigators have access to the final dataset. Data and analysis codes are available upon request from the corresponding author by email (csbang@hallym.ac.kr).
